# Air-Depleted and Solvent-Impregnated Cork Powder as a New Natural and Sustainable Fining Agent for Removal of 2,4,6-Trichloroanisole (TCA) from Red Wines

**DOI:** 10.3390/molecules27144614

**Published:** 2022-07-19

**Authors:** Fernanda Cosme, Sara Gomes, Alice Vilela, Luís Filipe-Ribeiro, Fernando M. Nunes

**Affiliations:** 1 Chemistry Research Centre-Vila Real (CQ-VR), Food and Wine Chemistry Lab, Department of Biology and Environment, School of Life Sciences and Environment, University of Trás-os-Montes and Alto Douro, 5000-801 Vila Real, Portugal; fcosme@utad.pt (F.C.); avimoura@utad.pt (A.V.); 2 Chemistry Research Centre-Vila Real (CQ-VR), Food and Wine Chemistry Lab, School of Life Sciences and Environment, University of Trás-os-Montes and Alto Douro, 5000-801 Vila Real, Portugal; sara_aras94@hotmail.com (S.G.); fmota@utad.pt (L.F.-R.); 3 Chemistry Research Centre-Vila Real (CQ-VR), Food and Wine Chemistry Lab, Chemistry Department, School of Life Sciences and Environment, University of Trás-os-Montes and Alto Douro, 5000-801 Vila Real, Portugal

**Keywords:** 2,4,6-Trichloroanisole (TCA), wine, ADSI cork powder, fining agent, phenolic profile, chromatic characteristics, volatile profile

## Abstract

Trichloroanisole (TCA) in wine results in a sensory defect called “cork taint”, a significant problem for the wine industry. Wines can become contaminated by TCA absorption from the atmosphere through contaminated wood barrels, cork stoppers, and wood pallets. Air-depleted solvent-impregnated (ADSI) cork powder (CP) was used to mitigate TCA in wines. The ADSI CP (0.25 g/L) removed 91% of TCA (6 ng/L levels), resulting in an olfactory activity value of 0.14. A Freundlich isotherm described ADSI CP TCA adsorption with irreversible adsorption and a K_F_ = 33.37. ADSI CP application had no significant impact on the phenolic profile and chromatic characteristics of red wine. Using headspace sampling with re-equilibration, an average reduction in the volatile abundance of 29 ± 15%, 31 ± 19%, and 37 ± 24% was observed for the 0.10, 0.25, and 0.50 g/L ADSI CP, respectively. The alkyl esters and acids were the most affected. The impact observed was much lower when using headspace sampling without re-equilibration. Isoamyl acetate, ethyl hexanoate, ethyl hexanoate, and ethyl decanoate abundances were not significantly different from the control wine and 0.25 g/L ADSI CP application. Thus, ADSI CP can be a new sustainable fining agent to remove this “off-flavor” from wine, with a reduced impact on the wine characteristics.

## 1. Introduction

2,4,6-Trichloroanisole (TCA) is a fungal metabolite with an unpleasant moldy odor that can contaminate wine, producing the so-called “cork taint” or “corked taste”. The “corked taste” is usually a musty, moldy, mildew, or earthy smell and is sometimes described as burnt rubber, smoke, or even camphor [1]. Other chloroanisoles, such as 2,4-dichloroanisole, 2,6-dichloroanisole, 2,3,4,6-tetrachloroanisole (TeCA), and pentachloroanisole (PCA), may also contribute to the “cork taste” but do not play a dominant role in this sensory defect. 2,4,6-Tribromoanisole (TBA) may also play a significant role in wine’s musty/mold odor [2]. TCA can be produced by different metabolic pathways. However, the formation of TCA from 2,4,6-trichlorophenol (TCP) by biomethylation reactions is the only scientifically proven origin. This biomethylation reaction is carried out through the enzyme chlorophenol *O*-methyltransferase, which is present in filamentous fungi of different families (*Streptomyces* spp., *Aspergillus* spp., *Trichoderma* spp., *Penicillium* spp. and *Cephalouscus* spp., among others). These fungi grow on different materials, such as cork and wood. Under high humidity and limited ventilation conditions, fungi can transform odorless chlorophenols with a high threshold of perception into chloroanisols with a low perception threshold. This enzyme catalyzes the reaction that converts halophenols into haloanisols [3]. Wine can be contaminated by TCA or other haloanisols even before it is bottled if it comes into contact with contaminated materials (such as wood barrels) and/or cellars that have a contaminated atmosphere [2]. According to Sefton and Simpson [4], the proportion of affected bottles is estimated to be between 1 and 5% and occasionally up to 30%. Contamination with chlorophenols caused by fungicides or insecticides can involve woody materials used for building cellars, wooden pallets for bottles, paints, boxes, and other materials such as polluted bottles, corks, and wines. TCA has also been identified as a contaminant of oak barrels [5].

TCA has an extremely low detection threshold of nanograms per liter (ng/L), which indicates that it will be easily detectable by the consumer, even at low concentrations. According to several authors, the sensory threshold of TCA in wine ranges from 1.4 ng/L to 4 ng/L [6,7,8,9,10,11], with values found in the literature that differ from author to author; for example, Vestner et al. [12] report that the sensory limit of TCA is around 4 ng/L (in wine). In contrast, Juanola et al. [13] refer to a 5 ng/L sensory threshold. Sefton and Simpson [4] mention that the detection limit can be between 1.4 and 4.6 ng/L and the recognition limit between 4.2 and 10 ng/L. On the other hand, Fontana et al. [14] state that the threshold of perception of TCA is greater than 0.03 ng/L. However, the threshold value in wine strongly depends on the type of wine, the wine’s style, and the taster’s experience [15].

Due to the sensory impact of TCA on wine, and the fact that TCA does not only originate from cork stoppers, it is necessary to find an effective technological solution that can eliminate or minimize the TCA in the wine with a minimal impact on its characteristics. A patent describes using an aqueous suspension of activated carbon obtained from coconut to remove the “cork taste” [16]. Another patent proposes contacting wine with synthetic aliphatic polymers (ultra-high molecular weight polyethylene) to reduce the TCA concentration. According to the data described in the patent, the TCA concentration of the treated wine is reduced from about 10 (ng/L) to preferably less than 5 (ng/L) or less, with the taste and smell of TCA in wine being undetectable below these values [17]. Vuchot et al. [18] used highly absorbent yeast cell extracts. Yeast cells were able to remove TCA (27%), TeCA (55%), and PCA (73%) without analytical or sensory modification of the wines. Doubling the dose yielded better results, allowing for a reduction by 45%, 73%, and 83%, respectively [18]. Molecularly imprinted polymers are synthetic materials with artificially generated recognition sites capable of specifically rebinding a target molecule. Molecularly imprinted polymers and non-molecularly imprinted polymers have been used with good results in wines for TCA removal with about 90% TCA removal [19].

The latest European Union legislation (EU Regulation 2019/934) allows for a filter plate treatment that contains Y-faujasite zeolites solely to adsorb haloanisols and is applied during filtration to reduce the concentration of the haloanisols responsible for flavor in wines below the threshold of perception. This treatment must be carried out on clarified wines, and the filter plates must be cleaned and disinfected before passing the wine through them and applying Y-faujasite zeolites [20].

Paraffin wax can absorb chloroanisols from wine, and absorption by polyethylene film can be even more effective, but TeCA was removed more efficiently than TCA. Polyethylene film offers an inexpensive and effective means of reducing trichloroanisole in wines, with only a slight impact on their characteristics. However, a loss of floral/fruity aroma was observed [21].

A plastic film composed of a mixture of synthetic polymers and certified for food use (where there is no migration of plastic molecules to the wine) was added to the wine at a dose of 20 m^2^ film/hL to study its efficiency in the removal of TCA from wine [22]. The removal of TCA from wine became more noticeable as the film–wine contact time increased. In barrels with contamination of 3 ng/L, the TCA concentration decreased by 47% after 8 h of treatment with the film. A more extended treatment of 24 h and 48 h led to a 73% and 83% reduction in TCA concentration, respectively. Furthermore, according to the results of this study, it can be observed that, globally, the use of plastic film to eliminate/reduce the content of haloanisols in wines did not impact the content of phenolic compounds (proanthocyanidins and anthocyanins) for up to 24 h of treatment with the film [22]. Valdés et al. [23] also studied the possibility of applying two polyaniline-based materials (100 to 500 mg/L) to remove TCA and TBA in methanol at a concentration of 20 ng/L. The results of these authors showed that the removal percentages of TCA and TBA were 68–72% and 84–85%, respectively, for the two materials tested in methanol, and their effectiveness varied with the interaction time and with the amount of polymer used.

Cork residues and cork powders have been used as bio-adsorbents to remove pesticides and other pollutants from wastewater with promising results [24]. This by-product obtained from the cork industry is an abundant, natural, and cheap material recently exploited in its raw form and after optimizing its adsorption properties by simple physicochemical treatments, such as air removal and simultaneous impregnation with ethanol. This treatment makes the cell wall components more accessible, demonstrating an increase of at least 4 times its adsorption capacity after treatment, which could be a new sustainable fining agent for wines [25,26]. This by-product was a good solution for the removal of volatile phenols without affecting the wine quality and sensory profile [25,26]. The use of cork dust waste produced in the cork stopper industry can increase its economic value and thus reduce the entry of new materials into the wine production chain. Due to its improved adsorption properties, air-depleted solvent-impregnated (ADSI) cork powder has a similar potential to other wine fining agents. The different adsorption mechanisms driven by hydrophobicity represent an alternative solution to be employed [25,26].

Therefore, this work aimed to study the efficiency of ADSI cork powder in the removal of TCA from red wines and the impact of its application on red wine characteristics, namely the chromatic characteristics, phenolic composition, and volatile profile.

## 2. Results and Discussion

### 2.1. Performance of Air-Depleted and Solvent-Impregnated Cork Powder in the Removal of Trichloroanisole (TCA)

The hydrophobic cork extractives were first removed by sequential treatment with dichloromethane and ethanol to increase the performance of natural cork powder in terms of its ability to remove TCA from the wine, as described by Filipe Ribeiro et al. [25]. As raw cork material contains significant amounts of trapped air, and water has a very low diffusion coefficient in cork, the air from the extracted cork powder was removed and impregnated with ethanol under vacuum by repeated degassing cycles (11 times) immersed in ethanol [25]. It was then sieved to obtain a particle size below 75 μm.

Wines were contaminated with two levels of TCA (3 and 6 ng/L). The treatment of the contaminated wines with air-depleted and solvent-impregnated cork powder at different doses (0.1, 0.25, and 0.5 g/L) decreased the wine’s TCA concentration significantly (Table 1). It was also observed that the higher the amount of ADSI cork powder applied to the wine, the greater its effectiveness in reducing the wine’s TCA concentration. Table 1 shows the percentage of TCA removal after the application of ADSI cork powder. There was observed an increase in the percentage of TCA removal with the increase in the applied dose of ADSI powder, and, as expected, the higher the concentration of TCA in wines the higher the removal percentage. Additionally, shown in Table 1 is the odor activity value (OAV) of TCA in the wines treated with ADSI cork powder. The OAV is a measure of the importance of a specific compound to the odor of the sample [27]. The odor detection threshold of TCA in wines varies widely in the literature, ranging from 1.4 to 22 ng/L depending on the study and also on the wine matrix. A more recent study using different white and red wine matrixes established a detection threshold of 4 and 5 ng/L of TCA both for aroma and flavor, respectively, while for 3 ng/L it was not considered significant; therefore, a detection threshold of 4 ng/L was used for calculating the OAV [15]. For all the application doses of ADSI cork powder for both TCA contamination levels, the OAV was well below 1 (Table 1); therefore, the impact of TCA on the aroma of wines treated with ADSI cork powder is expected to be negligible.

When compared with other research works that studied the removal of TCA from wine using different materials, it can be concluded that ADSI cork powder is one of the most effective materials for TCA removal. For example, with the application of highly absorbent yeast cell extract (0.4 g/L) added to wine containing 6 ng/L TCA, the removal was 27% of TCA, and doubling the application dose of yeast cell extract achieved better removal results (45%) [18]. For the use of molecularly imprinted polymers and non-molecularly imprinted polymers, good results were obtained (a TCA removal percentage of about 90%) [19]. Some plastics quickly absorb chloroanisols, and the absorption efficiency increases with the increase in the number of chlorine atoms in the molecule. Chloroanisols are hydrophobic substances and are therefore particularly soluble in non-polar media. Absorption of chloroanisoles from wine contaminated by non-polar substances such as food-grade paraffin wax or food-grade polyethylene film could be a viable way to reduce or even remove the odor of trichloroanisole from wine. Thus, the use of polyethylene film described by Capone et al. [21] showed that, after 4 days, it removed 90% of the TCA and 97% of the TeCA from white wine artificially contaminated with 100 ng/L of TCA and 100 ng/L of TeCA, respectively.

Valdes et al. [23] also showed that the application of two polyaniline-based materials (0.1 to 0.5 g/L) to wine contaminated with TCA and TBA (20 ng/L) had TCA and TBA removal percentages of 68–72%, and 84–85%, respectively. A recent study of the application of plastic film to wines stored in wooden barrels with 3 ng/L and 9 ng/L of TCA contamination showed that immersion of plastic film in wine for 8 h reduced the TCA concentration by 47% to 57%, and that after 24 h the TCA reductions were 73% and 75%, respectively. After 48 h of treatment, TCA concentration reductions of 83% and 81% were observed [22].

The results obtained in the present work using ADSI cork powder show that it was possible to remove 91% of the TCA with 0.25 g of ADSI cork powder/L of wine with an initial contamination of 6 ng/L of TCA (Table 1), which indicates that, compared with the other materials described in the literature, it is one of the most effective treatments in the removal of TCA from contaminated wines.

### 2.2. TCA Adsorption Isotherms of Air-Depleted Solvent-Impregnated Cork Powder in Model Wine

**Figure 1:**
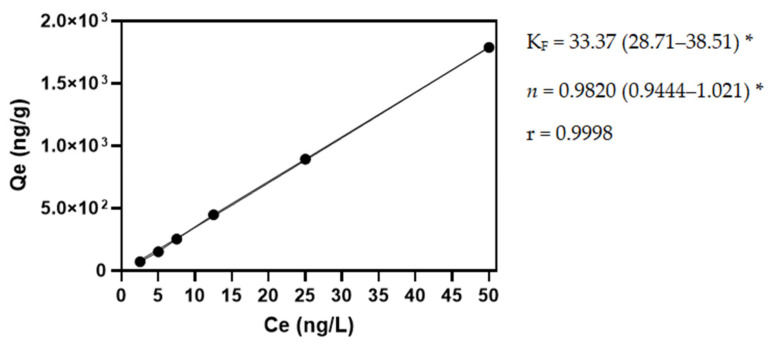
Freundlich adsorption isotherm of ADSI cork powder for TCA in a model wine solution. Qe is the amount of TCA adsorbed at equilibrium; Ce is the equilibrium concentration; * denotes the 95% confidence interval.

The adsorption isotherm of TCA to the ADSI cork powder was determined in a model wine solution at 25 °C for a 0.25 g/L application dose. As shown in Figure 1, the ADSI cork powder adsorption capacity increased in the entire concentration range assayed (2.5–50 ng/L of TCA in the model wine solution). To analyze the equilibrium data obtained experimentally, three isothermal models were used to characterize the adsorption system: the Langmuir, Freundlich, and Langmuir–Freundlich isotherm models [28]. The Langmuir isotherm is usually used for ideal monolayer adsorption on a homogeneous surface [29]. The Freundlich isotherm is generally suitable for nonideal adsorption on heterogeneous surfaces. It assumes that there are large numbers and many different types of available sites acting simultaneously, each with a different free energy of sorption [30]. Only the Freundlich model yielded high correlation coefficients (>0.999). The type of Freundlich isotherm is indicated by the value of *n*, in which both the K_F_ and *n* parameters are dependent on temperature. The 1/*n* value is the intensity of the adsorption or surface heterogeneity and indicates the energy distribution and the adsorbate sites’ heterogeneity. When 1/*n* is greater than zero (0 < 1/*n* < 1), the adsorption is favorable; when 1/*n* is greater than 1, the adsorption process is unfavorable, and it is irreversible when 1/*n* = 1 [31,32,33]. Therefore, the adsorption of TCA on ADSI cork powder seems to be irreversible, showing a K_F_ of 33.37.

### 2.3. Impact of ADSI Cork Powder on Wine Quality

To obtain a deeper insight into the impact of ADSI cork powder on the wine’s chemical composition, besides its TCA removal efficiency, the effects on the phenolic composition, chromatic characteristics, and volatile profile of the wine after application of increasing doses of ADSI cork powder were determined.

#### 2.3.1. Impact of ADSI Cork Powder on the Chromatic Characteristics and Phenolic Composition of the Wine

Table 2 shows the total phenolic compounds, color intensity, hue, and chromatic characteristics of red wines after the application of increasing doses of ADSI cork powder (0.10, 0.25, and 0.5 g/L). It can be observed that there are no significant differences in total polyphenols after the application of the ADSI cork powder compared with the control wine.

Gonzàlez-Centeno et al. [22], using plastic film to remove TCA, found that this material had little impact on the total phenolic compounds of the wine, with only a slight decrease (4.4%) in the total phenolic compounds concerning the untreated wine after 48 h of contact with the plastic film. In addition, the application of yeast cell extract at a dose of 400 mg/L for TCA removal did not significantly decrease the red wine’s color intensity [18].

The application of the ADSI cork powder did not significantly alter the color intensity and hue of the red wine (Table 2). These results agree with Filipe-Ribeiro et al. [25], who applied ADSI cork powder in red wine to remove volatile phenols and observed that the ADSI cork powder did not change the color intensity of red wines significantly. In line with the results obtained for the color intensity and hue, there were no significant changes in the chromatic characteristics of the wine (Table 2). These results agree with those obtained by Filipe-Ribeiro et al. [25], who applied ADSI cork powder to remove volatile phenols from red wines and also did not observe significant changes in the chromatic characteristics compared with the control wine.

In the wine treated with plastic film for the removal of TCA as described by Gonzàlez-Centeno et al. [22], the chromatic characteristics were not altered after the treatment of the wine in contact with the plastic film. Although there were significant differences between untreated and plastic-film-treated wines and even between plastic-film-treated wines with different contact times, these differences were not visually perceived by any taster during the sensory analysis.

Table 3 shows the total pigments, polymeric pigments, small polymeric pigments (SPPs), large polymeric pigments (LPPs), monomeric anthocyanins, and tannins of the red wine treated with ADSI cork powder for TCA removal. The data clearly show no significant impact on these wine parameters after application of the ADSI cork powder compared with the control wine.

After applying plastic film to remove TCA from red wine, Gonzàlez-Centeno et al. [22] observed that the total proanthocyanidin values remained constant regardless of the film–wine contact time. The results of anthocyanins in this study show that wines treated with plastic film exhibited a small but significant increase in the total anthocyanin concentration, both after 48 h and after 24 h of contact with the plastic film. This increase suggests that the plastic wrap can absorb certain compounds in wine that anthocyanins combine with. Additionally, Gonzàlez-Centeno et al. [22] showed that using plastic film to eliminate/reduce the TCA content in wines did not significantly affect their levels of proanthocyanidins and anthocyanins for up to 24 h of treatment with film or plastic film.

These results indicate that ADSI cork powder has a low impact on the phenolic profile of red wine. The content of individual phenolic acids and catechin did not show significant differences after applying the different doses of ADSI cork powder, except for the ethyl ether of coumaric acid, which showed a significant decrease (Table 4). These data agree with those obtained by Filipe-Ribeiro et al. [25], who also observed few significant changes in phenolic acids and catechin compared with untreated wine.

The data on monomeric anthocyanin levels are shown in Table 5. Generally, no significant differences were observed, except for malvidin-3-*O*-glucoside. However, the total monomeric anthocyanins did not show significant differences from untreated wine. These data also agree with those obtained by Filipe-Ribeiro et al. [25], who, when applying ADSI cork powder to red wine, observed few significant differences in the monomeric anthocyanin profiles of wines treated with ADSI cork powder compared with the untreated wine.

In the use of plastic film for the removal of TCA described by Gonzàlez-Centeno et al. [22], the duration of the plastic film treatment did not lead to significant differences between the plastic-film-treated wines regarding monomeric anthocyanins. However, compared with untreated wine (the control), plastic-film-treated wines had slightly higher concentrations of some monomeric anthocyanins after 8 h of contact with the plastic film (2–14%), with malvidin-3-*O*-glucoside and delphinidin-3-*O*-glucoside the main compounds responsible for these increases. These observations agree with what was previously described for total anthocyanins. They could be explained by the potential absorption by the plastic film of certain carbonyl compounds that tend to combine with anthocyanins. This absorption of anthocyanins by the ADSI cork powder was not verified in the present study.

#### 2.3.2. Impact of ADSI Cork Powder on Wine Volatile Composition

**Figure 2:**
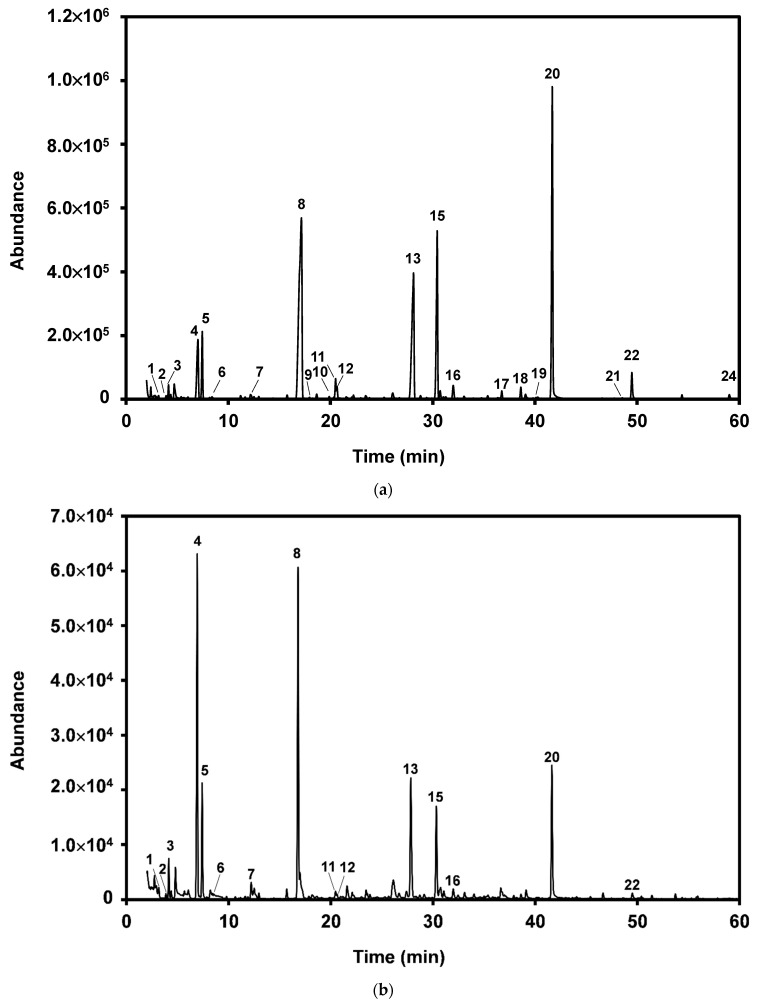
Typical chromatograms of red wines without ADSI cork powder addition using SPME headspace sampling with re-equilibration (**a**) and without re-equilibration (**b**). Only the major peaks are highlighted. For peak identification, refer to Table 6.

We used two methods of SPME headspace sampling to study the impact of the application of ADSI cork powder on the volatile profile of red wine. A standard lengthy steady-state extraction method, in which the extraction time allows for the re-equilibration of volatiles between the liquid matrix, headspace volatile, and SPME fiber, was used to extract the maximum amount of analyte. A fast snapshot method, whose reduced extraction time avoids/diminishes the re-equilibration of the headspace volatile composition above the wine, was also used without agitation and heating. Roberts and coworkers [34] found that HS-SPME with a short sampling time can determine the “true headspace” concentration at equilibrium between the headspace and water, which can minimize the disruption caused by the fiber/headspace partition. The “true headspace” discussed by Roberts et al. [34] reflects the volatile compounds in the air space at equilibrium between the headspace and the sample solution. Figure 2a and Table 6 show the volatile profile of red wines after applying three different doses of ADSI cork powder (0.1, 0.25, and 0.5 g/L), analyzed by two methods: headspace extraction with and without re-equilibration. When re-equilibration was allowed, the volatile abundance decreased with the increase in the ADSI cork powder dose applied. Even for the lowest dose of ADSI cork powder, there was observed a decrease in the abundance of almost all compounds analyzed, except for isoamyl alcohol, 3-methylbutanoic acid, diethylbutanoate, benzyl alcohol, phenylethanol, and decanoic acid (Table 6). For the 0.1 g/L ADSI cork powder application dose, an average reduction of 29 ± 15% was observed. The decline increased to 31 ± 19% and 37 ± 24% for the 0.25 g/L and 0.50 g/L ADSI cork powder application doses, respectively. The alkyl esters and acids were the most affected, resulting in average reductions of 48 ± 20% for the highest application dose. These results agree with those described by Filipe-Ribeiro et al. [25], who used ADSI cork powder for the removal of volatile phenols and observed a decrease in the total abundance of volatile compounds in the headspace with an increasing application dose of ADSI cork powder.

The use of the fast extraction method without re-equilibration, as expected, decreased the total abundance of the compounds extracted to only 4.46% (Table 6) but also changed the relative abundance of the extracted volatile compounds (Figure 2b and Table 6). When using this headspace sampling method, with few exceptions, significant reductions in the headspace volatile abundance were only significant for the 0.50 g/L ADSI cork powder application dose. For *p*-cymene, 3-methylbutanoic acid, 1,1,6-trimethyl-1,2-dihydronaphatalene, phenylethylacetate, phenylethanol, β-caryophyllene oxide, ethyl hexanoate, and decanoic acid, we observed a reduction in the headspace abundance with the application dose. A decrease in the abundance below the method detection limit for the less-abundant volatiles, such as phenylethylacetate, β-caryophyllene oxide, ethyl hexadecanoate, and decanoic acid, was also observed. Interestingly, for the low-molecular-weight alkyl esters, such as isoamyl acetate, ethyl hexanoate, ethyl hexanoate, and ethyl decanoate, the abundance observed for the 0.25 g/L ADSI cork powder application dose was not significantly different from that of the control wine. Therefore, although a substantial impact was observed on the abundance of the volatile compounds when headspace sampling with re-equilibration was employed, the apparent impact of ADSI cork powder application on the “true headspace” composition seems to be lower.

Compared with plastic film for TCA removal, for the longest contact time, Gonzàlez-Centeno et al. [22] observed an 82% reduction for ethyl octanoate, ethyl decanoate, and ethyl dodecanoate.

## 3. Materials and Methods

### 3.1. Cork Powder Sample Preparation

Cork powder with an average granulometry of 372 µm was obtained from a local cork stopper producer free of TCA and supplied by SAI. Lda. (Paredes, Portugal). To extract the extractives, the natural cork powder was subjected to a dichloromethane extraction by soxhlet for 24 h, followed by a second extraction with ethanol by soxhlet for 24 h. To obtain extractive-free cork powder with a particle size of less than 75 µm, the cork powder was sieved through a sieve. To remove the air contained in the cork powder and simultaneously impregnate the material with ethanol, proportions of 0.01 g, 0.025 g, and 0.05 g of cork powder were immersed in 5 mL of ethanol, and the suspension was vacuum-degassed (0.00131 atm) by repeated cycles (11 times). The number of degassing cycles was chosen by observing the sedimentation of the cork powder at the bottom of the container. After impregnation, the cork powder was left in contact with ethanol (96% *v*/*v*) for 12 h. After this period, the ethanol was removed by centrifugation for 10 min at 10.956 g and 20 °C. The ADSI cork powder was used for the wine fining experiments [25].

### 3.2. Wine Contamination with TCA

A red wine from the Douro region (vintage 2019) was used, with an alcohol content of 13.0 (% *v*/*v*), a total acidity of 5.4 g/L of tartaric acid, a volatile acidity of 0.38 g/L of acetic acid, and a pH of 3.70. Six liters of wine were divided into three parts (2 L each), in which one part was artificially contaminated with 3 ng/L of TCA, another part with 6 ng/L of TCA, and a third part was not contaminated with TCA, which was used as a control wine. These contamination levels were chosen by taking into account the “consumer rejection threshold” of 3.1 ng/L of TCA as described by Prescott et al. [8]. The free sulfur dioxide in the wine was adjusted to 50 mg/L.

### 3.3. Fining Experiment 

To study the performance of the cork powder in removing TCA, red wine samples were spiked with 3 ng/L and 6 ng/L of TCA. Different doses of cork powder (0 g, 0.10 g, 0.25 g, and 0.50 g) were added to 1 L of contaminated wine. The wine was left in contact with the cork powder for 6 days at room temperature, without stirring. After 6 days, the wine was centrifuged for 10 min at 10.956 g and 20 °C for analysis. All experiments were performed in duplicate.

### 3.4. Determination of 2,4,6-Trichloroanisole Extractable by Solid-Phase Microextraction (SPME) Using Gas Chromatography Coupled to Mass Spectrometry (GC-MS)

To determine 2,4,6-trichloroanisole, we used a 10 mL wine sample containing 3 g of NaCl and 100 μL of internal standard solution. D5-TCA (2 μg/L) was placed in 20 mL SPME vials, which were immediately sealed. Samples were analyzed using a GC-MS instrument equipped with an autosampler configured in SPME mode. The flasks were incubated for 2 min and extracted for 8 min, under agitation (250 rpm) at 50 °C, using a 100 μm PDMS fiber. The fiber was desorbed in the injector at 270 °C for 4 min in splitless mode. Compounds were separated on a 5 MS capillary column (30 m × 0.25 mm × 0.25 µm). The detection and quantification limits of this method are 0.2 ng/L and 0.5 ng/L, respectively. This analysis was carried out in cooperation with the company Souto & Castro. All analyses were performed in duplicate.

### 3.5. Quantification of Total Phenolic Compounds

The wine’s total phenolic compounds were determined using the absorbance at 280 nm according to Ribéreau-Gayon et al. [43]. The results are expressed as gallic acid equivalents through calibration curves with standard gallic acid. All analyses were performed in duplicate.

### 3.6. Color Intensity, Hue, and Chromatic Characteristics

The red wine’s color intensity and hue were quantified as described in the OIV methods [44]. For the chromatic characteristics of red wine, the absorption spectra of wine samples were scanned from 380 to 780 nm using a 1 cm path length quartz cell, and the wine’s chromatic characteristics (L* (lightness), a* (redness), and b* (yellowness) coordinates) were calculated using the International Commission on Illumination (CIE) method using the L*, a*, and b* coordinates according to the OIV [44]. The chroma (C* = [(a*)^2^ + (b*)^2^]^1/2^]) and hue-angle (h° = tang^−1^(b*/a*)) values were also determined. To distinguish the color more accurately, the color difference was calculated using the following equation: ΔE* = [(ΔL*)^2^ + (Δa*)^2^ + (Δb*)^2^]^1/2^. This parameter allows for the reliable quantification of the overall color difference in a sample compared to a control sample (untreated wine). Analyses were performed in duplicate.

### 3.7. High-Performance Liquid Chromatography (HPLC) Analysis of Anthocyanins, Catechin, and Phenolic Acids

Analyses were carried out with an Ultimate 3000 Dionex HPLC system equipped with a PDA-100 photodiode array detector (Dionex. Sunnyvale, CA, USA) and an Ultimate 3000 Dionex pump. The separation was performed on a C18 column (250 mm × 4.6 mm, 5 μm particle size, ACE, Aberdeen, Scotland) with a 1 mL/min flow rate at 35 °C. The injection volume was 50 μL, and the detection was performed in the wavelength range of 200 to 650 nm. The analysis was carried out using 5% aqueous formic acid (A) and methanol (B), and the gradient was as follows: 5% B from zero to 5 min, followed by a linear gradient up to 65% B until 65 min and from 65 to 67 min down to 5% B [45]. Quantification was performed with calibration curves with caffeic acid, coumaric acid, ferulic acid, gallic acid, and catechin as standards. *trans*-Caftaric acid, 2-*S*-glutathionylcaftaric acid (GRP), and caffeic acid ethyl ester are expressed as caffeic acid equivalents, and coutaric acid and coumaric acid ethyl ester are expressed as coumaric acid equivalents. A calibration curve of malvidin-3-glucoside, peonidin-3-glucoside, and cyanidin-3-glucoside was used to quantify these anthocyanins. Using the coefficient of molar absorptivity (ε) and extrapolation, it was possible to obtain the slopes for delphinidin-3-glucoside, petunidin-3-glucoside, and malvidin-3-coumaroylglucoside to perform the quantification. The results on delphinidin-3-acetylglucoside, petunidin-3-acetylglucoside, peonidin-3-acetylglucoside, cyanidin-3-acetylglucoside, and cyanidin-3-coumaroylglucoside are expressed as the respective glucoside equivalent [46,47].

### 3.8. Total Pigments, Polymeric Pigments, Small Polymeric Pigments (SPPs), Large Polymeric Pigments (LPPs), Anthocyanins, and Tannins

For profiling, the phenolic fractions responsible for the red wine color, the method described by Adams et al. [48] was used. This method combines the protein precipitation (BSA) assay and the bisulfite bleaching assay to distinguish monomeric anthocyanins from polymeric pigments, and two classes of polymeric pigments in wines can also be measured: small polymeric pigments (SPPs) that do not precipitate with proteins and large polymeric pigments (LPPs) that precipitate with proteins. The combination of SPPs and LPPs is equivalent to the sulfur-dioxide-resistant pigments in wine. In the first tube, 500 μL of wine was mixed with 1 mL of acetic acid–NaCl buffer (200 mM acetic acid and 170 mM NaCl, adjusted to pH 4.9 with sodium hydroxide). The absorbance at 520 nm (in a 1 mm path length cuvette) of the mixture was measured (A value). Then, 80 μL of a 0.36 M potassium metabisulfite solution was added. After 10 min of incubation, the absorbance at 520 nm was measured again (B value). The absorbance due to monomeric pigments can be calculated as (A-B), where the Β value represents the total amount of polymeric pigment (SPPs + LPPs). In a second tube, 500 μL of wine was mixed with 1 mL of acetic acid–NaCl buffer containing bovine serum albumin (BSA) (1 mg/mL). The mixture was allowed to stand at room temperature for 15 min with slow stirring, and then the tube was centrifuged for 5 min at 13.500 g to sediment the tannin–protein precipitate. One milliliter of the supernatant was mixed with 80 μL of a 0.36 M potassium metabisulfite solution. After 10 min of incubation, the absorbance at 520 nm was measured (C value). This absorbance (the C value) corresponds to the polymeric pigment that did not precipitate with the tannin and the protein. The absorbance is due to small polymeric pigments (SPPs), and this C value was used to calculate the amount of polymeric pigment that precipitated with the tannin and the protein (B-C) absorbance due to large polymeric pigments (LPPs). Total polymeric pigments (PPs) are the sum of the small polymeric pigments and the large polymeric pigments. The supernatant from the second experiment described above was discarded, and the remaining pellet was washed with 250 µL of acetic acid–NaCl buffer to remove residual monomeric anthocyanins. The tube was centrifuged for 1 min at 13.500 g, and the supernatant was discarded. Then, the pellet was dissolved in 875 μL of buffer containing 5% (*v*/*v*) triethanolamine (TEA) and 5% (*w*/*v*) sodium dodecyl sulfate (SDS). The buffer dissolves the precipitate containing tannins, proteins, and any polymeric pigments that precipitated with the tannin and the protein. After incubation, the tube was vortexed to dissolve any remaining precipitate. The absorbance at 510 nm (in a 10 mm path length cuvette) was measured after allowing the solution to stand at room temperature for 10 min (value D). To calculate the tannin absorbance, 125 μL of a ferric chloride solution was added (10 mM ferric chloride and 10 mM hydrochloric acid in water). The absorbance at 510 was reread after 10 min (value E). All analyses were performed in duplicate.

### 3.9. Wine Volatile Composition Determined by SPME-GC-MS

Two methods were used to analyze the volatile profile of wines, namely headspace extraction with and without re-equilibration [30].

To determine the headspace volatile composition of red wines with re-equilibration, a validated method was confirmed in our laboratory [49]. Briefly, the Divinylbenzene/Carboxen/Polydimethylsiloxane (DVB/CAR/PDMS) 50/30 μm fiber was conditioned before use by insertion into the GC injector at 270 °C for 60 min. To a 20 mL headspace vial, we added 10 mL of wine and 2.5 g of NaCl. The vial was sealed with a Teflon septum. The fiber was inserted through the vial septum previously conditioned at 35 °C and exposed for 60 min with agitation to perform the extraction by an automatic CombiPal system. The fiber was inserted into the injection port of the GC for 3 min at 270 °C. All analyses were performed in duplicate.

To determine the headspace volatile composition of red wines without re-equilibration, the extraction time was initially evaluated by measuring the headspace abundance and profile after extraction during 1, 2, and 3 min. The abundance of the obtained chromatograms increased as the extraction time increased. As the relative abundance of the peaks did not change significantly between 1 and 3 min, the extraction time of 3 min was used. The Divinylbenzene/Carboxen/Polydimethylsiloxane (DVB/CAR/PDMS) 50/30 μm fiber was conditioned before use by insertion into the GC injector at 270 °C for 60 min. To a 20 mL headspace vial, 10 mL of wine was added. The vial was sealed with a Teflon septum. The fiber was inserted through the vial septum previously conditioned at 25 °C (room temperature) and exposed for 3 min without agitation to perform the extraction by an automatic CombiPal system. The fiber was inserted into the injection port of the GC for 3 min at 270 °C. All analyses were performed in duplicate.

Analyses were performed by gas chromatography using a Trace GC Ultra system with a Polaris Q mass spectrometer. Separation was performed using a DB-FFAP column (30 m × 0.25 mm, and 0.25 μm film thickness) with a 1 mL/min helium flow. The oven temperature program was: 40 °C for 5 min, increased to 155 °C at 5 °C/min, then increased to 300 °C at 20 °C/min, and held at that temperature for 1 min. All analyses were performed in duplicate.

### 3.10. Modeling of the Adsorption Isotherms

After determining the amount of cork powder that best removed TCA (0.025 g), a model wine solution was prepared (ethanol at 12.0% *v*/*v* with 3.5 g/L of tartaric acid; the pH of the solution was adjusted to 3.60 with NaOH). A total of 0.025 g of ADSI cork powder was placed per 100 mL of model wine solution, and an increasing concentration of TCA (2.5 ng/L, 5 ng/L, 7.5 ng/L, 12.5 ng/L, 25 ng/L, and 50 ng/L) was used. Three isothermal models were used to characterize the adsorption systems, namely the Langmuir, Freundlich, and Langmuir–Freundlich isotherm models, to analyze the equilibrium data obtained experimentally. The Langmuir model is the simplest and the most frequently used in adsorption studies. This model assumes that adsorption occurs on a homogeneous surface with identical active sites and uniform energies [28]. In the Langmuir model, the Langmuir isotherm expression is represented by the following equation [28]:

Q_e_ = (Q_max_ × K_L_ × C_e_)/(1 + K_L_ × C_e_) (1)

where KL is the Langmuir constant related to the affinity of the active sites, Q_max_ is the theoretical maximum monolayer capacity, C_e_ is the equilibrium concentration, and Q_e_ is the amount of TCA adsorbed at equilibrium.

The Freundlich model assumes that adsorption occurs on a heterogeneous surface with an exponential distribution of active sites and energies [28], and it is expressed by the equation:

Q_e_ = K_F_ × C_e_^1/*n*^ (2)

where K_F_ is the Freundlich constant, and C_e_ and Q_e_ are defined as above and related to the adsorption favorability and adsorption capacity, respectively.

The Freundlich constant (K_F_) is related to the adsorption capacity, and the constant *n* is related to the adsorption intensity. Values of *n* in the range 1 < *n* < 10 indicate favorable adsorption.

The Langmuir–Freundlich isotherm—also known as the Sips equation—is capable of modeling homogeneous and heterogeneous bonding surfaces and is expressed by [50]:

Q_e_ = (Q_m_ × K_s_ × C_e_^n^)/(1 + (K_s_ × C_e_^n^) (3)

where Q_e_ and C_e_ are described as above, Q_m_ is the total number of binding sites, and *n* represents the system’s heterogeneity index, which can vary from 0 to 1. If *n* = 1, the system is homogeneous and can be equated to the Langmuir model, and *n* < 1 represents a heterogeneous material. K_s_ is a parameter related to the median binding affinity (K_0_) via K_0_ = a1/*n*, where *n* is the heterogeneity index, which ranges from 0 to 1.

The Langmuir–Freundlich isotherm is composed of the Langmuir isotherm and the Freundlich isotherm and can be reduced to either one in its limits. When *n* = 1, the Langmuir–Freundlich isotherm reduces to the Langmuir isotherm, which corresponds directly to the binding affinity (K_L_). Alternatively, as Ce or a approaches 0, the Langmuir–Freundlich isotherm reduces to the Freundlich isotherm. Furthermore, the Langmuir–Freundlich isotherm reduces to the Freundlich isotherm for all systems at low concentrations.

### 3.11. Statistical Treatment

Statistically significant differences between means were determined by analysis of variance (ANOVA, one-way) followed by Tukey’s honestly significant difference (HSD, 5% level) post-hoc test for the physicochemical data. All analyses were performed using Statistica 10 software (StatSoft, Tulsa, OK, USA).

## 4. Conclusions

The application of air-depleted solvent-impregnated cork powder in a 0.25 g/L dose to red wine contaminated with TCA (6 ng/L) resulted in a significant decrease in TCA levels (a 91% reduction). Applying ADSI cork powder up to 0.50 g/L did not result in a significant change in the red wine’s phenolic composition and chromatic characteristics. On the other hand, the application of ADSI cork powder resulted in a significant decrease in the red wine’s volatile composition when determined by exhaustive headspace extraction. However, the impact on the “true headspace” concentration was much lower. This natural material may represent a new and efficient technological solution with a low environmental impact, contributing to a more sustainable wine industry. 

## Figures and Tables

**Table 1:** TCA remaining in wine contaminated with 3 ng/L of TCA and 6 ng/L of TCA after applying different doses of ADSI cork powder (0.10, 0.25, and 0.50 g/L) and the corresponding TCA odor activity values (OAVs) in the final wines.

Wine	TCA Remaining (ng/L)	OAV
Wine with 3 ng/L of TCA		
0.10 g/L	2.25 ± 0.35 ^a^	0.56
0.25 g/L	1.95 ± 0.25 ^a^	0.49
0.50 g/L	1.35 ± 0.25 ^a^	0.34
Wine with 6 ng/L of TCA		
0.10 g/L	3.30 ± 0.40 ^a^	0.83
0.25 g/L	0.55 ± 1.05 ^a^	0.14
0.50 g/L	1.40 ± 0.30 ^a^	0.35

Values in the same column for each contamination level (3 ng/L TCA or 6 ng/L TCA) followed by the same letter are not significantly different (Tukey’s HSD, *p* ≤ 0.05).

**Table 2:** Total phenolic compounds, color intensity, hue, and chromatic characteristics of red wines after the application of different doses of ADSI cork powder (0.10, 0.25, and 0.50 g/L).

Wine	Total Phenolic Compounds (mg/L)	Color Intensity a.u.	Hue	L*	a*	b*	C*	h°	ΔE*
Control	1544 ± 187 ^a^	15.02 ± 0.24 ^a^	0.71 ± 0.00 ^a^	70.1 ± 0.5 ^a^	35.05 ± 0.96 ^a^	7.06 ± 0.15 ^a^	35.75 ± 0.96 ^a^	0.20 ± 0.01 ^a^	-
0.10 g/L	1694 ± 263 ^a^	15.14 ± 0.53 ^a^	0.71 ± 0.02 ^a^	69.6 ± 0.7 ^a^	35.21 ± 1.95 ^a^	7.08 ± 0.49 ^a^	35.92 ± 2.00 ^a^	0.20 ± 0.01 ^a^	1.79 ± 1.11 ^a^
0.25 g/L	1425 ± 199 ^a^	14.79 ± 0.07 ^a^	0.71 ± 0.00 ^a^	70.2 ± 0.7 ^a^	34.21 ± 0.31 ^a^	7.06 ± 0.13 ^a^	35.04 ± 0.32 ^a^	0.20 ± 0.00 ^a^	1.45 ± 0.33 ^a^
0.50 g/L	1513 ± 224 ^a^	14.83 ± 0.29 ^a^	0.71 ± 0.01 ^a^	70.1 ± 0.7 ^a^	34.21 ± 0.31 ^a^	7.11 ± 0.35 ^a^	34.94 ± 0.36 ^a^	0.20 ± 0.01 ^a^	1.11 ± 0.64 ^a^

L* (lightness), a* (redness), b* (yellowness) coordinates, C* (chroma), h° (hue-angle), ΔE* (total color difference in relation to control wine). Values in the same column followed by the same letter are not significantly different (*n* = 8) (Tukey’s HSD, *p* ≤ 0.05). a.u. (Absorbance unit).

**Table 3:** Total pigments, polymeric pigments, small polymeric pigments (SPPs), large polymeric pigments (LPPs), monomeric anthocyanins, and tannins of red wines after applying different doses of ADSI cork powder (0.10, 0.25, and 0.50 g/L).

Wine	Total Pigments a.u.	Polymeric Pigments (SPPs + LPPs) a.u.	SPPs a.u.	LPPs a.u.	Monomeric Anthocyanins a.u.	Tannins a.u.
Control	5.27 ± 0.09 ^a^	2.54 ± 0.05 ^a^	1.53 ± 0.05 ^a^	1.00 ± 0.08 ^a^	2.74 ± 0.04 ^a^	0.81 ± 0.47 ^a^
0.10 g/L	5.29 ± 0.09 ^a^	2.54 ± 0.06 ^a^	1.67 ± 0.30 ^a^	0.87 ± 0.31 ^a^	2.75 ± 0.04 ^a^	0.87 ± 0.03 ^a^
0.25 g/L	5.26 ± 0.15 ^a^	2.52 ± 0.08 ^a^	1.51 ± 0.07 ^a^	1.01 ± 0.10 ^a^	2.74 ± 0.07 ^a^	0.80 ± 0.24 ^a^
0.50 g/L	5.38 ± 0.23 ^a^	2.59 ± 0.10 ^a^	1.51 ± 0.14 ^a^	1.08 ± 0.16 ^a^	2.79 ± 0.14 ^a^	1.15 ± 0.75 ^a^

Values in the same column followed by the same letter are not significantly different (*n* = 8) (Tukey’s HSD, *p* ≤ 0.05). a.u. (Absorbance unit).

**Table 4:** Phenolic acid profile and flavonoids in mg/L of red wines after applying different doses of ADSI cork powder (0.10, 0.25, and 0.50 g/L).

Wine	Catechin	Gallic Acid	*trans*-Caftaric Acid	Coutaric Acid Isomer	Coutaric Acid	Caffeic Acid	*p*-Coumaric Acid	Ferulic Acid	Ethyl Ester of Caffeic Acid	Ethyl Ester of Coumaric Acid
Control	7.36 ± 1.43 ^a^	20.34 ± 0.49 ^a^	5.57 ± 0.20 ^a^	6.56 ± 0.14 ^a^	2.20 ± 0.28 ^a^	3.16 ± 0.15 ^a^	1.53 ± 0.08 ^a^	0.76 ± 0.12 ^a^	0.37 ± 0.01 ^a^	1.66 ± 0.06 ^b^
0.10 g/L	7.73 ± 1.14 ^a^	20.63 ± 0.24 ^a^	5.53 ± 0.38 ^a^	6.46 ± 0.73 ^a^	1.90 ± 0.43 ^a^	3.18 ± 0.16 ^a^	1.56 ± 0.24 ^a^	0.96 ± 0.43 ^a^	0.36 ± 0.03 ^a^	1.48 ± 0.11 ^a^
0.25 g/L	8.29 ± 0.93 ^a^	20.31 ± 0.35 ^a^	5.76 ± 0.05 ^a^	6.64 ± 0.24 ^a^	2.20 ± 0.34 ^a^	3.10 ± 0.24 ^a^	1.68 ± 0.14 ^a^	0.82 ± 0.14 ^a^	0.37 ± 0.03 ^a^	1.49 ± 0.19 ^b^
0.50 g/L	8.02 ± 0.65 ^a^	20.65 ± 0.21 ^a^	5.48 ± 0.39 ^a^	6.45 ± 0.63 ^a^	2.22 ± 0.37 ^a^	3.02 ± 0.20 ^a^	1.47 ± 0.17 ^a^	0.70 ± 0.04 ^a^	0.36 ± 0.01 ^a^	1.52 ± 0.10 ^ab^

Values in the same column followed by the same letter are not significantly different (*n* = 4) (Tukey’s HSD, *p* ≤ 0.05).

**Table 5:** Monomeric anthocyanin profile in mg/L of red wines after applying different doses of ADSI cork powder (0.10, 0.25, and 0.50 g/L).

Wine	D-3-G	C-3-G	Pet-3-G	Peo-3-G	M-3-G	D-3-A	Pet-3-A	Peo-3-A	M-3-A	C-3-C	M-3-C	Total Monomeric Anthocyanins
Control	1.11 ± 0.06 ^a^	4.65 ± 0.35 ^a^	6.88 ± 0.34 ^a^	5.56 ± 0.11 ^a^	32.22 ± 0.43 ^a^	0.28 ± 0.03 ^a^	0.46 ± 0.04 ^a^	0.08 ± 0.16 ^a^	4.27 ± 0.56 ^a^	0.38 ± 0.03 ^a^	5.15 ± 0.37 ^a^	61.05 ± 0.72 ^a^
0.10 g/L	1.04 ± 0.17 ^a^	4.46 ± 0.17 ^a^	6.96 ± 0.18	5.55 ± 0.38 ^a^	32.10 ± 1.75 ^a^	0.24 ± 0.16 ^a^	0.51 ± 0.21 ^a^	0.08 ± 0.16 ^a^	4.18 ± 0.48 ^a^	0.36 ± 0.06 ^a^	4.79 ± 0.96 ^a^	60.27 ± 1.20 ^a^
0.25 g/L	1.01 ± 0.14 ^a^	4.65 ± 0.16 ^a^	6.78 ± 0.31 ^a^	5.59 ± 0.34 ^a^	31.98 ± 1.08 ^a^	0.21 ± 0.15 ^a^	0.51 ± 0.11 ^a^	0.18 ± 0.20 ^a^	4.26 ± 0.34 ^a^	0.35 ± 0.07 ^a^	5.17 ± 0.20 ^a^	60.69 ± 1.40 ^a^
0.50 g/L	0.98 ± 0.12 ^a^	4.32 ± 0.25 ^a^	6.70 ± 0.14 ^a^	5.30 ± 0.60 ^a^	31.92 ± 1.29 ^a^	0.22 ± 0.17 ^a^	0.59 ± 0.14 ^a^	0.23 ± 0.17 ^a^	4.55 ± 0.18 ^a^	0.42 ± 0.12 ^a^	5.24 ± 0.28 ^a^	60.46 ± 1.89 ^a^

Delphinidin-3-*O*-glucoside (D-3-G), Cyanidin-3-*O*-glucoside (C-3-G), Petunidin-3-*O*-glucoside (Pet-3-G), Peonidin-3-*O*-glucoside (Peo-3-G), Malvidin-3-*O*-glucoside (M-3-G), Delphinidin-3-*O*-acetylglucoside (D-3-A), Petunidin-3-*O*-acetylglucoside (Pet-3-A), Peonidin-3-*O*-acetylglucoside (Peo-3-A), Malvidin-3-*O*-acetylglucoside (M-3-A), Cyanidin-3-*O*-coumaroylglucoside (C-3-C), Malvidin-3-*O*-coumaroylglucoside (M-3-C). Values in the same column followed by the same letter are not significantly different (*n* = 4) (Tukey’s HSD, *p* ≤ 0.05).

**Table 6:** Volatile components identified in the red wine headspace by SPME extraction with re-equilibration and without re-equilibration and the effect of ADSI cork powder application dose (0.10, 0.25, and 0.50 g/L) on the volatile abundance (area × 10^5^).

Peak	Compound	RI	Aroma Descriptors	With Re-Equilibration				Without Re-Equilibration			
				Control	0.10 g/L	0.25 g/L	0.50 g/L	Control	0.10 g/L	0.25 g/L	0.50 g/L
1	Hexanal **	1083	Green, woody, vegetative, apple, grassy, citrus, and orange	0.19 ± 0.01 ^a^	0.13 ± 0.01 ^b^	0.14 ± 0.01 ^b^	0.13 ± 0.01 ^b^	0.038 ± 0.002 ^a^	0.034 ± 0.003 ^a,b^	0.029 ± 0.001 ^a,b^	0.027 ± 0.004 ^b^
2	Acetaldehyde ethyl amyl acetal **	1098		0.31 ± 0.02 ^a^	0.26 ± 0.00 ^a,b^	0.26 ± 0.00 ^a,b^	0.24 ± 0.01 ^b^	0.023 ± 0.000 ^a^	0.022 ± 0.001 ^a^	0.024 ± 0.000 ^a^	0.021 ± 0.003 ^a^
3	Isoamyl acetate *	1144	Banana	5.09 ± 0.47 ^a^	3.67 ± 0.42 ^b^	3.37 ± 0.12 ^b^	3.16 ± 0.24 ^b^	1.02 ± 0.07 ^a^	1.16 ± 0.04 ^a^	1.01 ± 0.04 ^a^	0.71 ± 0.02 ^b^
4	Isoamyl alcohol *	1194	Alcohol, floral, cheese	40.6 ± 4.8 ^a^	34.1 ± 1.1 ^a^	33.9 ± 0.3 ^a^	32.0 ± 1.7 ^a^	7.89 ± 0.25 ^a^	8.19 ± 0.07 ^a^	8.26 ± 0.13 ^a^	8.06 ± 0.02 ^a^
5	Ethyl hexanoate *	1203	Fruity, strawberry, green apple, anise	29.7 ± 3.4 ^a^	23.5 ± 0.4 ^a,b^	22.2 ± 0.4 ^b^	20.2 ± 0.6 ^b^	2.90 ± 0.07 ^a^	3.05 ± 0.06 ^a^	3.07 ± 0.09 ^a^	2.86 ± 0.03 ^a^
6	*p*-Cymene **	1223	Fruity, sweet	0.20 ± 0.01 ^a^	0.17 ± 0.00 ^b^	0.15 ± 0.00 ^c^	0.12 ± 0.01 ^d^	0.030 ± 0.001 ^a^	0.024 ± 0.001 ^b^	0.025 ± 0.000 ^b^	0.019 ± 0.001 ^c^
7	Hexanol *	1340	Green grass	0.21 ± 0.0 ^a^	0.18 ± 0.01 ^a,b^	0.17 ± 0.00 ^b^	0.17 ± 0.00 ^b^	0.031 ± 0.001 ^a^	0.031 ± 0.001 ^a^	0.029 ± 0.001 ^a^	0.029 ± 0.001 ^a^
8	Ethyl octanoate *	1418	Sweet, fruit, fresh, pineapple, pear, floral	320 ± 29 ^a^	248 ± 3 ^b^	220 ± 2 ^b^	190 ± 2 ^b^	10.4 ± 0.1 ^a^	10.3 ± 0.4 ^a^	9.88 ± 0.47 ^a,b^	8.76 ± 0.23 ^b^
9	Isopentyl hexnaoate **	1420	Fruity, banana, apple, pineapple, green	0.04 ± 0.00 ^a^	0.02 ± 0.01 ^a,b^	0.02 ± 0.00 ^a,b^	0.02 ± 0.00 ^b^	0.001 ± 0.000 ^a^	0.001 ± 0.000 ^a^	0.001 ± 0.000 ^a^	0.001 ± 0.000 ^a^
10	Terpinen-4-ol acetate **	1462	Peppery, woody, earthy, musty, sweet	0.21 ± 0.00 ^a^	0.19 ± 0.00 ^b^	0.17 ± 0.00 ^b^	0.15 ± 0.01 ^c^	0.003 ± 0.000 ^a^	0.003 ± 0.000 ^a^	0.002 ± 0.000 ^a,b^	0.002 ± 0.000 ^b^
11	Vitispirane A **	1475	Fruity, floral, earthy, woody, camphor, eucalyptus, spice	1.50 ± 0.10 ^a^	1.25 ± 0.00 ^b^	1.10 ± 0.00 ^b,c^	0.95 ± 0.02 ^c^	0.025 ± 0.000 ^a^	0.026 ± 0.001 ^a^	0.023 ± 0.000 ^b^	n.d. ^c^
12	Vitispirane B **	1487	Floral, camphor, eucalyptus, spice, wood	0.51 ± 0.03 ^a^	0.45 ± 0.00 ^a,b^	0.41 ± 0.01 ^b,c^	0.34 ± 0.01 ^d^	0.009 ± 0.000 ^a^	0.010 ± 0.000 ^a^	0.009 ± 0.001 ^a^	n.d.^b^
13	Ethyl decanoate *	1625	Grape, pleasant, soap	153 ± 16 ^a^	105 ± 3 ^b^	80.9 ± 1.8 ^c,d^	57.0 ± 1.6 ^d^	6.65 ± 0.14 ^a^	6.47 ± 0.45 ^a^	5.35 ± 0.47 ^a,b^	4.48 ± 0.36 ^b^
14	3-methylbutanoic acid **	1672	Cheese, fatty, rancid	0.07 ± 0.00 ^a^	0.07 ± 0.00 ^a^	0.07 ± 0.00 ^a^	0.07 ± 0.01 ^a^	0.006 ± 0.000 ^a^	0.004 ± 0.000 ^b^	0.004 ± 0.000 ^b,c^	0.003 ± 0.000 ^c^
15	Diethyl succinate *	1683	Fruity, apple, cooked apple, ylang	104 ± 4 ^a^	95.6 ± 0.6 ^a^	96.1 ± 5.2 ^a^	91.1 ± 9.1 ^a^	3.76 ± 0.21 ^a^	4.27 ± 0.06 ^b^	3.23 ± 0.02 ^c^	3.18 ± 0.04 ^c^
16	1,1,6-trimethyl-1,2-dihydronaphatalene (TDN) **	1716	Floral, fruit, pleasant,	8.59 ± 0.75 ^a^	5.02 ± 0.26 ^b^	3.66 ± 0.03 ^c,d^	2.70 ± 0.16 ^d^	0.59 ± 0.03 ^a^	0.50 ± 0.02 ^b^	0.46 ± 0.01 ^b,c^	0.42 ± 0.01 ^c^
17	Phenylethyl acetate *	1815	Roses, flowery	1.49 ± 0.00 ^a^	1.50 ± 0.23 ^a^	1.17 ± 0.10 ^a^	1.06 ± 0.04 ^a^	0.012 ± 0.001 ^a^	n.d.^b^	n.d.^b^	n.d.^b^
18	Ethyl dodecanoate *	1819	Flowery, fruity	7.31 ± 0.44 ^a^	3.76 ± 0.00 ^b^	2.21 ± 0.40 ^c^	1.17 ± 0.15 ^c^	0.39 ± 0.04 ^a^	0.32 ± 0.04 ^a,b^	0.19 ± 0.03 ^b^	0.19 ± 0.03 ^b^
19	Benzyl alcohol *	1885	Floral, citrusy, sweet	0.18 ± 0.01 ^a^	0.19 ± 0.00 ^a^	0.18 ± 0.00 ^a^	0.18 ± 0.03 ^a^	0.007 ± 0.000 ^a^	0.006 ± 0.000 ^a^	0.006 ± 0.000 ^a^	0.006 ± 0.001 ^a^
20	Phenylethanol *	1919	Roses, sweet	163 ± 3 ^a^	145 ± 9 ^a^	145 ± 4 ^a^	158 ± 24 ^a^	3.81 ± 0.28 ^a^	2.75 ± 0.16 ^b^	2.68 ± 0.28 ^b^	2.40 ± 0.02 ^b^
21	β-Caryophyllene oxide **	2005	Sweet, fresh, dry, woody, spicy	1.70 ± 0.05 ^a^	1.21 ± 0.10 ^b^	0.88 ± 0.01 ^c^	0.68 ± 0.03 ^c^	0.042 ± 0.001 ^a^	n.d.^b^	n.d.^b^	n.d.^b^
22	Octanoic acid *	2061	Fatty acid, rancid	10.4 ± 0.7 ^a^	6.44 ± 0.18 ^b^	5.97 ± 0.37 ^b^	5.71 ± 0.63 ^b^	0.25 ± 0.02 ^a^	0.21 ± 0.01 ^a^	0.12 ± 0.01 ^b^	0.13 ± 0.02 ^b^
23	Ethyl hexadecanoate *	2255	Fatty, rancid, fruity, sweet	0.06 ± 0.00 ^a^	0.03 ± 0.01 ^b^	0.03 ± 0.00 ^b^	0.02 ± 0.00 ^b^	0.006 ± 0.001 ^a^	n.d.^b^	n.d.^b^	n.d.^b^
24	Decanoic acid *	2281	Fatty, rancid, soap	3.35 ± 0.15 ^a^	2.82 ± 0.98 ^a^	1.89 ± 0.13 ^a^	1.54 ± 0.22 ^a^	0.10 ± 0.01 ^a^	n.d.^b^	n.d.^b^	n.d.^b^
25	Ethyl hydrogen succinate **	2378	Sweet, sour, fruity	2.24 ± 0.09 ^a^	1.99 ± 0.50 ^a,b^	1.10 ± 0.01 ^b^	2.10 ± 0.16 ^a,b^	0.096 ± 0.007 ^a^	0.071 ± 0.003 ^b^	0.056 ± 0.005 ^b^	n.d.^c^
26	Dodecanoic acid *	2464	Fatty, acidic, soapy, waxy	0.03 ± 0.00 ^a^	0.02 ± 0.01 ^a,b^	0.01 ± 0.01 ^a,b^	0.01 ± 0.00 ^b^	0.003 ± 0.001 ^a^	0.003 ± 0.000 ^a^	0.002 ± 0.000 ^a,b^	n.d. ^b^

Values are presented as the mean ± standard deviation (*n* = 2). Values in the same column for each headspace sampling method used followed by the same letter are not significantly different (One-way ANOVA, Tukey’s HSD post hoc test, *p* ≤ 0.05). RI, Kovats retention index. Odor descriptor from [35,36,37,38,39,40,41,42]. n.d., not detected. The reliability of the identification or structural proposal is indicated by the following: (*) mass spectrum and retention time consistent with those of an authentic standard; (**) structural proposals are given based on mass spectral data (Wiley 275) or are consistent with spectra found in the literature.

## Data Availability

Not applicable.
